# Comparing Continuous and Intermittent Exercise: An “Isoeffort” and “Isotime” Approach

**DOI:** 10.1371/journal.pone.0094990

**Published:** 2014-04-15

**Authors:** Andrea Nicolò, Ilenia Bazzucchi, Jonida Haxhi, Francesco Felici, Massimo Sacchetti

**Affiliations:** Department of Human Movement, Social and Health Sciences, “Foro Italico” University, Rome, Italy; University of Bath, United Kingdom

## Abstract

The present study proposes an alternative way of comparing performance and acute physiological responses to continuous exercise with those of intermittent exercise, ensuring similar between-protocol overall effort (isoeffort) and the same total duration of exercise (isotime). This approach was expected to overcome some drawbacks of traditional methods of comparison. Fourteen competitive cyclists (20±3 yrs) performed a preliminary incremental test and four experimental 30-min self-paced protocols, i.e. one continuous and three passive-recovery intermittent exercise protocols with different work-to-rest ratios (2 = 40∶20s, 1 = 30∶30s and 0.5 = 20∶40s). A “maximal session effort” prescription was adopted for this experimental design. As expected, a robust perceived exertion template was observed irrespective of exercise protocol. Similar between-protocol pacing strategies further support the use of the proposed approach in competitive cyclists. Total work, oxygen uptake and heart rate mean values were significantly higher (*P*<0.05) in the continuous compared to intermittent protocols, while lactate values were lower. Manipulating the work-to-rest ratio in intermittent exercise, total work, oxygen uptake and heart rate mean values decreased with the decrease in the work-to-rest ratio, while lactate values increased. Despite this complex physiological picture, all protocols showed similar ventilatory responses and a nearly perfect relationship between respiratory frequency and perceived exertion. In conclusion, our data indicate that overall effort and total duration of exercise are two critical parameters that should both be controlled when comparing continuous with intermittent exercise. On an isoeffort and isotime basis, the work-to-rest ratio manipulation affects physiological responses in a different way from what has been reported in literature with traditional methods of comparison. Finally, our data suggest that during intermittent exercise respiratory frequency reflects physiological strain better than oxygen uptake, heart rate and blood lactate.

## Introduction

The comparison of continuous (CON) with intermittent (INT) exercise is a central issue in sport science and exercise physiology. When low-volume INT is compared with CON, as well as when the two modes of exercise are compared on a work-matched basis, INT induces similar or even superior adaptations in a range of physiological, performance-related and health-related markers [1]. Moreover, INT is considered as being superior to CON in inducing further adaptations in already highly-trained endurance athletes [2].

Acute physiological responses to INT vs. CON have been extensively studied and traditionally compared on a work-matched basis [3]–[5]. However, work matching of training bouts when comparing CON and INT is highly inconsistent with descriptive data quantifying the actual training characteristics of successful endurance athletes across a broad range of sports [6]. Moreover, it induces unequal overall effort among different exercise protocols [7], [8]. Indeed, using this kind of comparison, previous studies have reported that INT is more physiologically demanding than CON, as indicated by higher values of oxygen uptake (VO_2_), heart rate (HR), minute ventilation (V_E_), blood lactate (La^−^) and rate of perceived exertion (RPE) [3]–[5]. Therefore, in order to ensure maximal physical stress in different exercise protocols (thus a comparable physiological demand), many authors have used time to exhaustion (TTE) protocols [9]–[13]. Nonetheless, apart from concerns about validity and reliability [14], a TTE protocol has the disadvantage of inducing high between-subject variability in TTE and hence heterogeneous physiological responses, both in CON [15] and in INT protocols [9]–[13]. Moreover, when comparing exercise protocols to exhaustion, considerably different total durations of exercise can be found [9], [11]–[13], which can mask the real between-protocol differences in physiological responses.

As a solution to the above-mentioned problems, Seiler and colleagues [8], [16], [17] introduced a novel approach, called “isoeffort”, for comparing various INT exercise protocols. This modality, extensively used to prescribe INT in athletic training [16], [17], requires athletes to self-pace their exercise intensity in response to a prescription of “maximal session effort” [8], ensuring similar between-protocol overall effort [17]. They used this approach to investigate performance and physiological responses to INT protocols with different work durations [17], with different recovery time [16], as well as adaptations to different intensity and total work duration training regimens [8].

However, to the best of our knowledge, the “isoeffort” approach has never been used to compare either CON to INT exercise, or various INT protocols with different work-to-rest ratios. This was the aim of the present study. We compared performance and physiological responses in four cycling protocols, one CON and three INT with different work-to-rest ratios. The protocols were designed on an “isoeffort” basis and matched for total duration of exercise (“isotime”). We expected this matching modality to overcome some drawbacks of traditionally used methods of comparison, and thus provide novel insight into acute physiological responses to CON vs. INT.

## Materials and Methods

### Ethics Statement

All participants gave their written informed consent according to the declaration of Helsinki. For minors enrolled in the study, we obtained written informed consent from the next of kin. The experimental protocols were approved by the Ethics Committee of the University of Rome La Sapienza.

### Subjects

Fourteen male subjects (mean ± SD: age 20±3 years, stature 1.76±0.07 m, body mass 64±7 kg) volunteered to participate in this study. All the subjects were well-trained [18] competitive cyclists with a minimum of 3 years’ cycling experience and 350 km training per week. The subjects were asked to refrain from strenuous exercise, consumption of alcohol and caffeine for at least 24 h before each test.

### Experimental Overview

All testing was completed in the laboratory with a room temperature of 20–21°C and at the same time of day (±2 h). Participants visited the laboratory on 5 occasions over a four-week period, with visits separated by at least 48 hours. In the first visit, subjects performed a preliminary ramp incremental exercise test. In the following visits, they performed four experimental cycling protocols, one CON and three INT protocols differing in the work-to-rest ratio. The experimental protocols were performed in a random order. All the cycling protocols were performed on an electromagnetically-braked cycle ergometer (Lode Excalibur Sport, Groningen, the Netherlands), whose settings were adjusted and recorded for each subject during the first visit to be reproduced in the following visits. Performance, physiological and perceptual responses were recorded during all the tests.

### Preliminary Test

Before the ramp incremental test, the Borg 6–20 Rating of Perceived Exertion (RPE) scale was presented to subjects, and appropriate instructions about the interpretation and use of the scale were given according to established recommendations [19]. During the test, subjects were asked to express an RPE value every minute during exercise and immediately after exhaustion. RPE data from this test served for familiarization purposes and were not used for further analysis.

A 5 min warm-up at 100W, 2 min of rest and 3 min of unloaded pedaling preceded the incremental ramp exercise test to exhaustion, which consisted of a continuous ramped increase in work rate of 30 W·min^−1^, starting from 0W. Preferred pedaling cadence (94±3 rpm) was selected by each subject and was kept constant throughout the test, which terminated when cadence fell by more than 10 rpm, despite strong verbal encouragement. During the test, pulmonary gas exchange was measured breath-by-breath as described below. VO_2_max was defined as the highest value of a 30-s average and the maximum power output of the test (Pmax) as the highest power output achieved at exhaustion, registered to the nearest 1 W. Breath-by-breath data were averaged over 10s and the first ventilatory threshold (VT1) was determined from a cluster of measures including 1) the first disproportionate increase in carbon dioxide output (VCO_2_) from visual inspection of individual plots of VCO_2_ versus VO_2_, 2) an increase in V_E_/VO_2_ with no increase in V_E_/VCO_2_, and 3) an increase in end-tidal O_2_ tension with no fall in end-tidal CO_2_ tension. The second ventilatory threshold (VT2) was determined from a cluster of measurements including 1) the first disproportionate increase in minute ventilation (V_E_) from visual inspection of individual plots of V_E_ versus VCO_2_, 2) the first systematic increase in V_E_/VCO_2_, and 3) the first systematic decrease in end-tidal CO_2_ tension [20]. The power output values corresponding to VT1 (PVT1) and VT2 (PVT2) were estimated with account taken of the mean response time of the VO_2_ response, which was assumed to approximate 40s [20].

After recovering from exercise, subjects were familiarized with the linear mode of the ergometer used in the experimental protocols. In this modality, also called rpm-dependent mode, torque is linearly related to cadence, while power output is exponentially related to cadence. Subjects were required not to increase pedaling cadence excessively (keeping it at about 100 rpm) immediately before starting the work phase of INT protocols, so as to avoid rpm-dependent artifacts in power output.

### Experimental Protocols

The experimental protocols, consisting of a CON and three INT protocols with different work-to-rest ratios (2 = 40∶20s, 1 = 30∶30s and 0.5 = 20∶40s), were matched for total duration of exercise (30 min). In all the protocols, subjects were required to self-select their exercise intensity in response to a prescription of “maximal session effort” [8]. This approach was used to ensure similar between-protocol overall effort [17]. Recovery in INT protocols consisted of unloaded pedaling (with the ergometer in the hyperbolic mode). In line with previous studies adopting the linear mode in cycling [21], the α linear factor (indicating the slope of the relationship between torque and cadence) was set for each subject considering the 50% Δ (the power output halfway between PVT1 and Pmax, expressed in W) and the preferred cadence, according to the formula: α = 50% Δ/preferred cadence^2^. The linear factor was kept constant for each subject in the 4 experimental protocols. Given the fact that power output was rpm-dependent, this would allow subject to pedal at higher cadences during INT protocols compared to CON exercise. The fact that the freely chosen cadence adopted by competitive cyclists during INT formats similar to those investigated in the present study [11] is higher than the cadence reported during a 30-min CON time trial [22], supports our decision to keep the linear factor constant for each subject.

Prior to the experimental protocols, participants performed a standardized warm up consisting of 3 min at 100 W, 6 min at 50% Pmax, 1 min at 60% Pmax, two self-paced 10s submaximal sprints with 40 s recovery in between, and finishing with 1 min of pedaling at 100W. The experimental protocols were preceded by 3 min of unloaded pedaling and started with the work phase.

With the exception of elapsed time, no feedback on performance or physiological measurements and no encouragement, either during or after the cycling protocol, was given to participants to minimize external factor influence [14]. Power output, heart rate (HR), gas exchange parameters, minute ventilation (V_E_) and respiratory frequency (f_R_) were registered continuously during the cycling experimental protocols, while La^−^ and RPE were collected every three minutes. Blood lactate was also collected before the experimental protocols and 3 min post exercise.

### Cardiopulmonary Parameters and Blood Lactate

Pulmonary gas exchange and ventilatory parameters were measured breath-by-breath using open-circuit indirect calorimetry (Quark b2, Cosmed, Rome, Italy). Appropriate calibration procedures were performed following the manufacturer’s instructions.

Capillary blood samples were drawn from the earlobe and lactate was measured by a portable lactate analyzer (Lactate Pro, Arkray KDK, Japan).

### Data Analysis

With the exception of power output, physiological parameters were averaged continuously during the intermittent exercise bouts and summarized as 3 min time epochs. Average power was calculated from work period only.

The maximum value of respiratory frequency (f_R_) was defined as the highest 60s average (including rest periods in INT) for all the five protocols performed in this study.

The relationship between RPE and f_R_ was calculated for all the experimental protocols. A correlation between these two parameters was also obtained pooling together data from the 4 experimental protocols. Using the regression equation of this correlation, f_R_ (expressed as a % of the f_R_max) was associated with the 15-point RPE scale.

### Statistical Analysis

Statistical analyses were carried out with PASW statistics 18 (SPSS Inc, Chicago, Illinois, USA). A one-way repeated-measures ANOVA was used to analyze mean values of performance and physiological parameters. A two-way repeated-measures ANOVA was used to analyze performance and physiological parameters as a function of time. When the sphericity assumption was violated, the Greenhouse-Geisser adjustment was performed. When significant differences were found, the Bonferroni test was used to determine the origin of such differences. Linear regressions with Pearson’s coefficient were used to establish the relationship between RPE/f_R_, RPE/elapsed time and f_R_/elapsed time respectively. Descriptors recommended by Hopkins [23] were used to express the magnitude of correlations. Alpha level was set at *P*<0.05 for all the statistical analyses performed. The results are expressed as mean (±SD).

## Results

The VO_2_max and the Pmax measured during the ramp incremental test were 4.3±0.3 L min^−1^ (67±6 ml kg^−1^ min^−1^) and 434±34 W, respectively. PVT1 and VO_2_ at VT1 were 213±24 W and 2.9±0.2 L min^−1^, respectively. PVT2 and VO_2_ at VT2 were 308±24 W and 3.7±0.2 L min^−1^, respectively.

**Figure 1:**
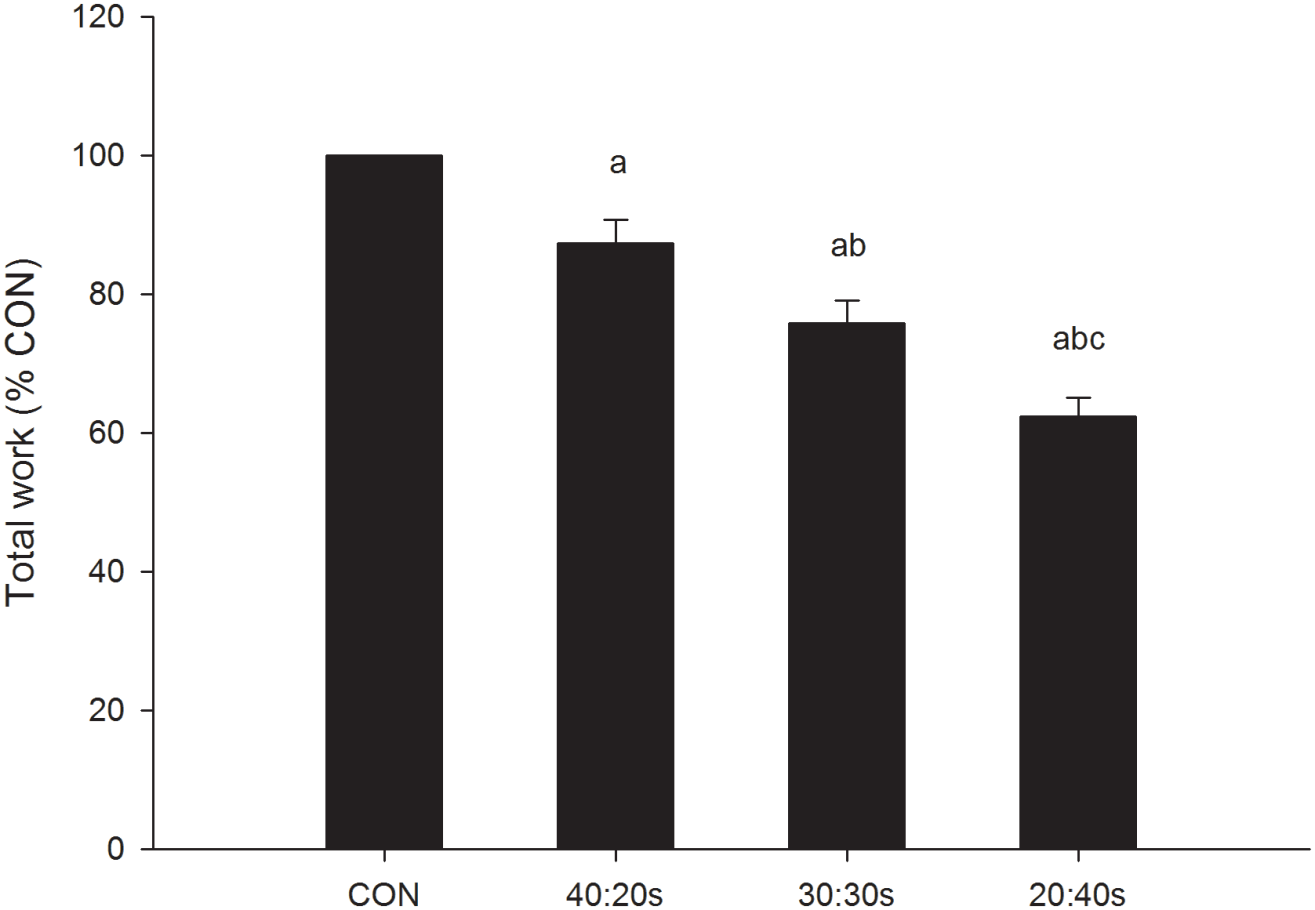
Total work. Total work expressed as a % of CON value, for the 4 experimental protocols. (a) *P*<0.05 vs. CON. (b) *P*<0.05 vs. 40∶20s. (c) *P*<0.05 vs. 30∶30s.

Table 1 reports mean values of several performance and physiological parameters for the 4 experimental protocols. Mean power output (considering only the work phase in INT protocols) was significantly different (*P*<0.05) in all cases (CON = 307±36, 40∶20s = 400±35, 30∶30s = 464±51 and 20∶40s = 573±66 W). Total work was higher in CON compared to all INT protocols (*P*<0.05). In the INT protocols, total work decreased with the decrease in the work-to-rest ratio, with differences observed in all cases (*P*<0.05). Values expressed relative to CON total work were 87±3%, 76±3% and 62±3% for 40∶20s, 30∶30s and 20∶40s, respectively (figure 1). Mean VO_2_ was higher in CON and lower in INT, particularly for INT protocols with a lower work-to-rest ratio, and all the protocols differed to one another (*P*<0.05), with the exception of the comparison between CON and 40∶20s, where a trend (*P* = 0.056) was observed (table 1). Conversely, an opposite situation was observed for La^−^, with the lowest values found in CON (5.8±2.5 mmol·L^−1^) and the highest values in the 20∶40s (9.5±2.2 mmol·L^−1^). Significant differences (*P*<0.05) were observed for CON and 40∶20s (7.2±2.7 mmol·L^−1^) compared to 20∶40s, and for 30∶30s (8.5±2.4 mmol·L^−1^) compared to CON. No between-protocol differences were found for mean values of f_R_ and V_E_ (table 1).

**Table 1:** Total work and mean values of several performance and physiological parameters for the four experimental protocols.

	CON	40∶20s	30∶30s	20∶40s
Total work (kJ)	552±65	480±42 a	418±46 ab	344±40 abc
Power output (W)	307±36	267±23 a	232±25 ab	191±22 abc
Power output of work phase (W)	307±36	400±35 a	464±51 ab	573±66 abc
Pedaling cadence (rpm)	90±4	101±4 a	104±5 ab	109±5 abc
VO_2_ (ml·min^−1^)	3655±382	3443±263	3147±234 ab	3000±307 abc
VO_2_ (%VO_2max_)	85±6	80±5	75±6 ab	69±5 abc
HR (beats·min^−1^)	174±4	173±7	169±5 ab	164±7 abc
HR (%HR_max_)	91±3	90±3	88±3 ab	85±4 abc
La^−^ (mmol·L^−1^)	5.8±2.5	7.2±2.7	8.5±2.4 a	9.5±2.2 ab
f_R_ (breaths·min^−1^)	46±4	46±5	45±6	45±6
V_T_ (L)	2.52±0.44	2.50±0.41	2.56±0.49	2.53±0.46
V_E_ (L·min^−1^)	115±23	115±17	113±17	112±19

VO_2_ =  oxygen uptake; HR  =  heart rate; La^−^  =  blood lactate; f_R_  =  respiratory frequency; V_T_  =  tidal volume; V_E_  =  minute ventilation.

Values are expressed as mean ± SD. If not otherwise specified, recovery phases in INT are included. (a) *P*<0.05 vs. CON. (b) *P*<0.05 vs. 40∶20s. (c) *P*<0.05 vs. 30∶30s.

**Figure 2:**
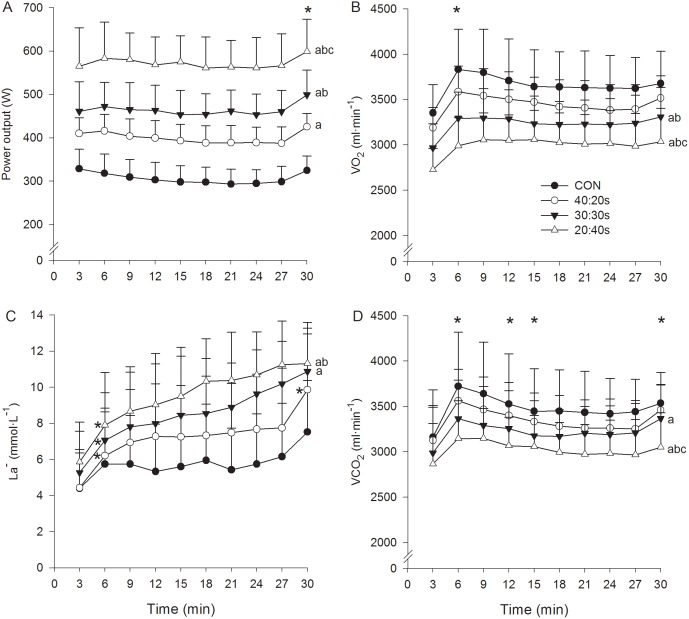
Power output, blood lactate and gas exchange parameters. Power output (work phase only in INT) (A), VO_2_ (B), blood lactate (C) and VCO_2_ (D) for CON (closed circles), 40∶20s (open circles), 30∶30s (closed triangles) and 20∶40s (open triangles). Data points for gas exchange parameters result from a three minute average, including also the recovery periods in INT. Letters (a, b and c) report main effect of protocol. (a) *P*<0.05 vs. CON. (b) *P*<0.05 vs. 40∶20s. (c) *P*<0.05 vs. 30∶30s. Asterisks in the upper part of each graph report main effect of time, while asterisks near a data point indicate protocol by time interaction (found for La^−^). *P<0.05 vs. the previous value.

**Figure 3:**
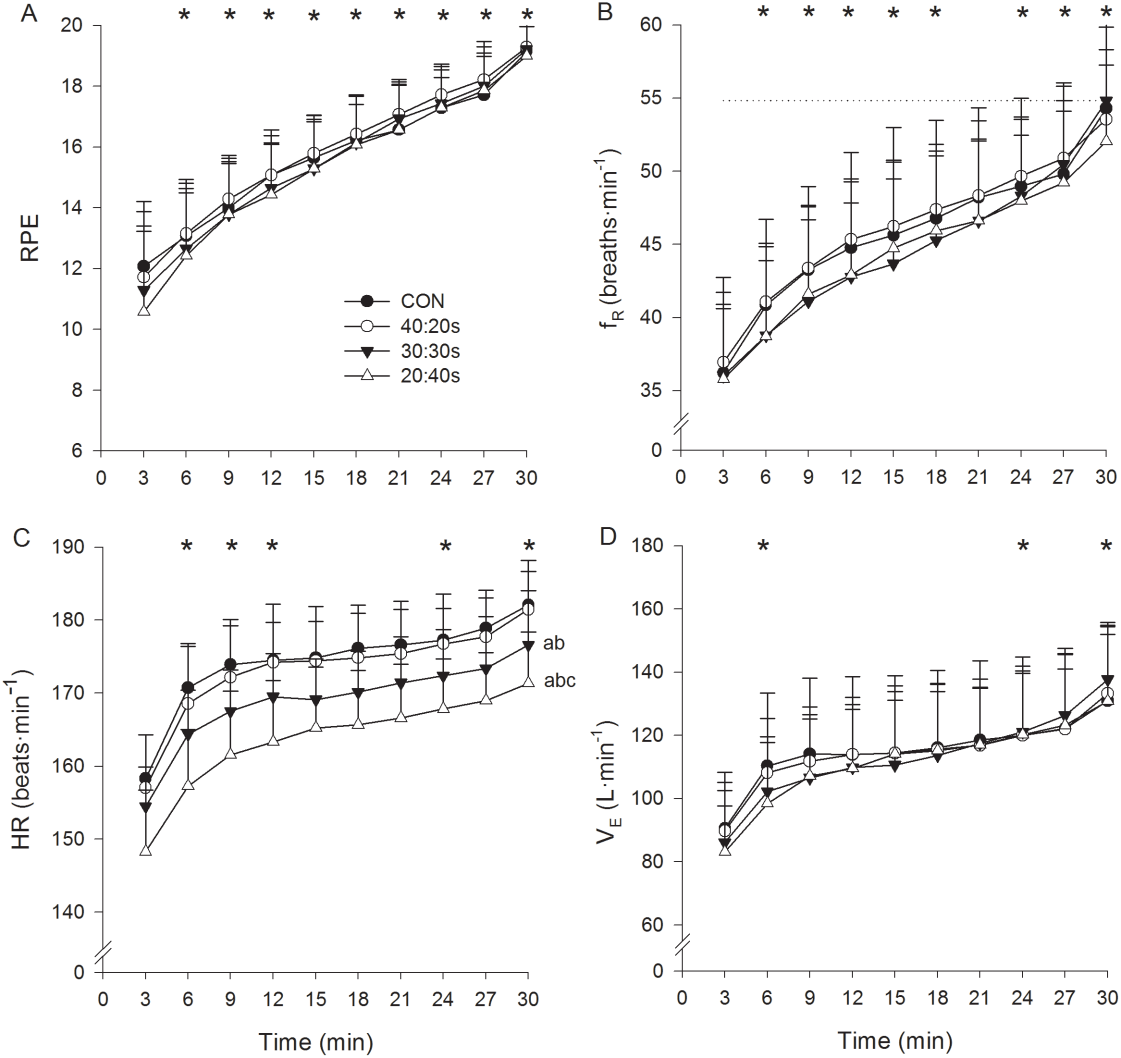
Perceived exertion, heart rate and respiratory parameters. RPE (A), f_R_ (B), HR (C) and V_E_ (D) for CON (closed circles), 40∶20s (open circles), 30∶30s (closed triangles) and 20∶40s (open triangles). Data points for f_R_, HR and V_E_ result from a three minute average, including also the recovery periods in INT. Dotted line in panel B indicate the f_R_ maximum value reached in the incremental ramp exercise test. Letters (a, b and c) report main effect of protocol. (a) *P*<0.05 vs. CON. (b) *P*<0.05 vs. 40∶20s. (c) *P*<0.05 vs. 30∶30s. Asterisks in the upper part of each graph report main effect of time. No protocol by time interaction was observed for the 4 variables. *P<0.05 vs. the previous value.

**Figure 4:**
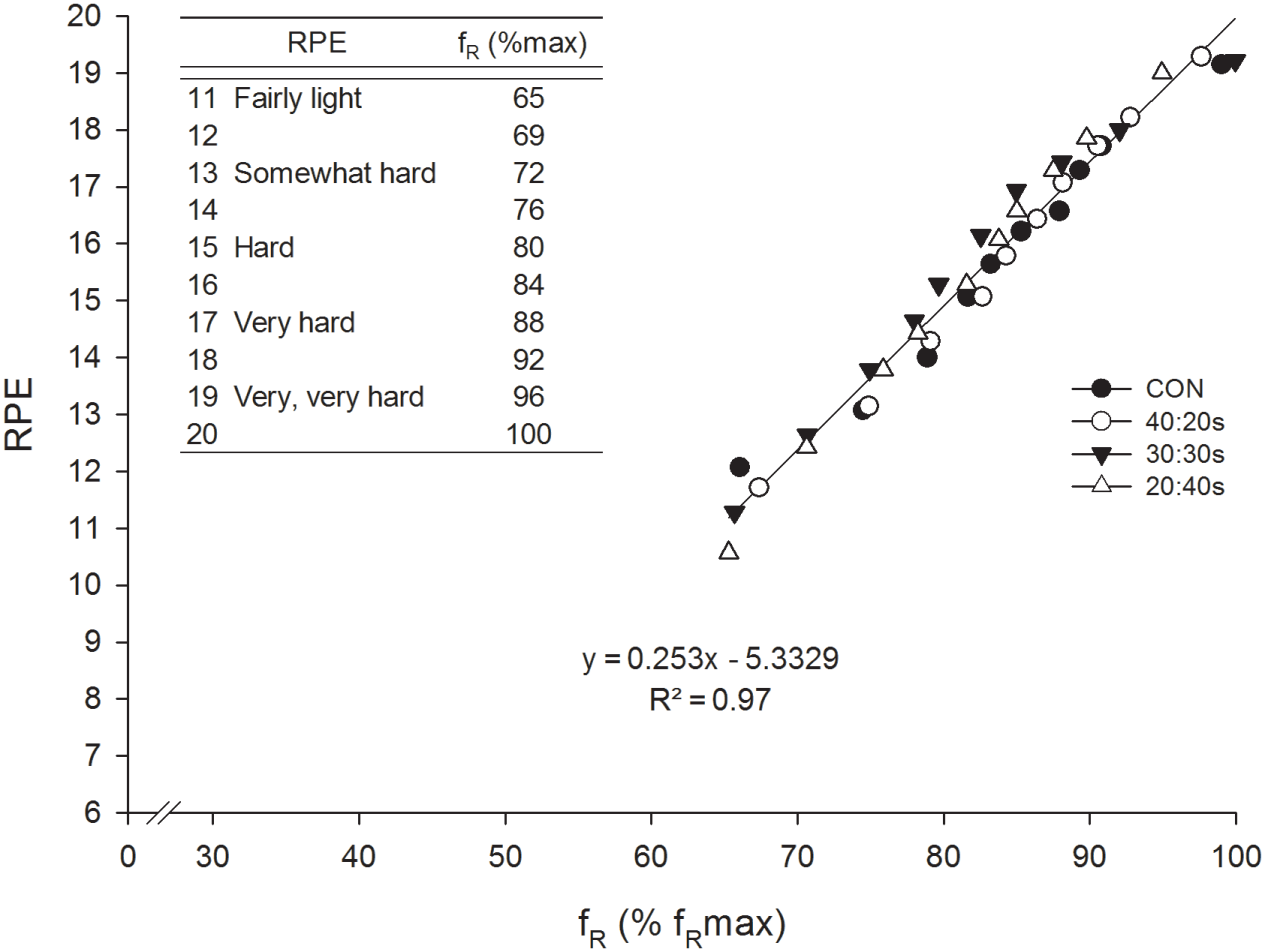
Relationship between perceived exertion and respiratory frequency. Relationship between RPE values and 3-min average values of f_R_, expressed as a % of the f_R_ maximum value reached in the ramp incremental exercise test, for CON (closed circles), 40∶20s (open circles), 30∶30s (closed triangles) and 20∶40s (open triangles). The linear regression results from pooling together data from the 4 experimental protocols. The regression equation of the relationship has been used to associate f_R_ with the 15-point RPE scale for the RPE range registered in this study (from 11 to 20), as shown in the upper left corner of the chart.

Figures 2 and 3 depict power output (considering only the work phase in INT), VO_2_, La^−^, VCO_2_, RPE, f_R_, HR and V_E_ as a function of time for the 4 experimental protocols. A significant protocol by time interaction was found for La^−^, where a more rapid increase of La^−^ at the onset of exercise was observed for the three INT protocols compared to CON. A main effect of time was observed for all the parameters investigated, and a main effect of protocol was found for power output, VO_2_, VCO_2_, La^−^ and HR, but not for f_R_, RPE and V_E_. In particular, power output data showed a similar pacing strategy among the 4 protocols, and a robust, almost linear growth over time was found for RPE and f_R_. Moreover, a significant (*P*<0.01), nearly perfect relationship was found between f_R_ and RPE for the 4 experimental protocols, as indicated by the correlation coefficients (CON = 0.91±0.08, 40∶20s = 0.92±0.05, 30∶30s = 0.93±0.07 and 20∶40s = 0.91±0.08). The relationship between f_R_ and RPE was also nearly perfect when pooling together values of the 4 protocols (figure 4). In addition, a significant (*P*<0.01) nearly perfect relationship was found between RPE and elapsed time for the 4 protocols (CON = 0.93±0.04, 40∶20s = 0.95±0.04, 30∶30s = 0.96±0.04 and 20∶40s = 0.93±0.04) and a significant (*P*<0.01) very large/nearly perfect correlation between f_R_ and elapsed time (CON = 0.87±0.11, 40∶20s = 0.89±0.10, 30∶30s = 0.92±0.05 and 20∶40s = 0.87±0.19). No differences in f_R_ maximum values were found between the 5 protocols performed in this study (ramp = 54.8±7.4, CON = 57.6±5.8, 40∶20s = 56.7±4.4, 30∶30s = 57.8±5.9 and 20∶40s = 54.8±5 breaths·min^−1^).

## Discussion

The present investigation proposes a new modality of comparing performance and physiological responses to continuous with those of intermittent exercise protocols, and highlights the importance of matching these protocols for both total duration of exercise and overall effort. On an “isoeffort” and “isotime” basis, the higher total work found in CON compared to INT indicates that in order to ensure similar between-protocol overall effort, the total work should be different in the two exercise modes. In addition, when manipulating the work-to-rest ratio with this approach we obtained a complex physiological picture that differs from the responses reported in literature with traditional methods of comparison [11], [24]–[26]. Within this physiological framework, similar between-protocol responses of respiratory frequency, as well as a nearly perfect relationship with RPE in all the experimental protocols indicate that, during INT exercise, respiratory frequency reflects physiological strain better than the physiological parameters traditionally used.

In order to remove both between-subject and between-protocol variability in time to exhaustion, typical of TTE protocols [9]–[13], in the present study the total duration of exercise was fixed for all the protocols investigated. We also required athletes to self-select their exercise intensity in response to a prescription of “maximal session effort” [8]. This approach, called “isoeffort” [8], has the great advantage of ensuring similar overall effort among exercise protocols [17], differently from the traditionally employed work-matching modality [8]. Developed on the basis of how INT exercise is prescribed in athletic training, the “isoeffort” approach has been adopted in only a few of the investigations focusing on INT [7], [8],[16],[17]. In particular, to the best of our knowledge, it had never been used to compare either CON to INT exercise, or INT protocols differing in the work-to-rest ratio, as we have done in the present study.

Since we constrained the total duration of exercise to 30 min, the mean exercise intensity during the CON protocol approximated the VT2 intensity. It should be acknowledged that this is generally not representative of continuous training sessions, which are often performed by athletes at lower exercise intensities [6]. However, the unique design used in our study provides novel insights into physiological responses to continuous and intermittent exercise protocols, with important implications for exercise prescription. It could be argued that the comparison of different self-paced “isotime” protocols can be influenced by the pacing strategy adopted, but our data add the novel finding that competitive cyclists adopt a similar pacing strategy with a robust RPE template, irrespective of exercise protocol. This confirms the effectiveness of this approach in inducing similar between-protocol overall effort in well-trained athletes. In this perspective, the reason why the study that first proposed the term “isoeffort” [8] did not show similar between-protocol RPE values, is probably due to the adoption of considerably different total durations of exercise in various INT protocols. Nevertheless, Seiler et al. [8] demonstrated that the “isoeffort” approach allows for targeting specific physiological responses, contrasting previous observations [27].

On an “isoeffort” and “isotime” basis, our data demonstrate that CON enables athletes to accumulate considerably greater total work compared to INT. This is direct evidence that in order to provide similar between-protocol effort, total work should be higher in CON than in INT. In other words, this suggests that, when comparing different exercise protocols on a work-matched basis, researchers should be aware that they are comparing a more demanding INT protocol with a less demanding CON protocol. This is of particular importance, considering that the large body of evidence demonstrating similar or even superior adaptations after INT compared to CON training, mainly arises from studies that compare the two modalities on a work-matched basis [1].

Our data showed higher VO_2_ values, but lower La^−^ values in CON compared to INT. In intermittent protocols, as the work-to-rest ratio decreased, the VO_2_ decreased as well, while the La^−^ increased. In contrast, previous studies that investigated the effect of work-to-rest ratio manipulation on physiological responses [11], [24]–[26] did not reveal an opposite trend in VO_2_ and La^−^ responses (high VO_2_– low La^−^ and viceversa). In fact, Ballor and Volovsek [24] compared three INT protocols with the same work and rest durations to those adopted in our study, with the difference that both the absolute work intensity and the total duration of exercise were fixed. They found that HR,V_E_,VO_2_ and blood lactate responses were higher in the 40∶20s, lower in the 20∶40s and in-between in the 30∶30s. The discrepancy with our results can therefore be explained by the fact that they matched for total duration of exercise but not for overall effort, comparing a highly demanding 40∶20s with a less demanding 30∶30s and an even less demanding 20∶40s. In a previous investigation [11], we also compared a 40∶20s with a 30∶30s INT with the same absolute exercise intensity, but the exercise was prolonged to exhaustion. In line with what is reported by Ballor and Volovsek [24], that study showed higher values of HR,V_E_,VO_2_ and blood lactate in the 40∶20s compared to the 30∶30s. On that occasion we matched for overall effort (maximal at the end of exercise), but the total duration of exercise was considerably longer in the 30∶30s. Collectively, the differences between the previous and the present data indicate that overall effort and total duration of exercise are two crucial variables that have to be both controlled when investigating acute physiological responses.

From a practical viewpoint, it is interesting to note that the parameters generally used to monitor exercise intensity or physiological strain during CON exercise, i.e. power output, HR, VO_2_ and La^−^ showed contrasting responses during INT exercise. Based on HR response alone which is the most used physiological parameter in the field [27], it could be concluded that as the work-to-rest ratio decreases the physiological demand of an exercise protocol decreases as well. Conversely, the analysis of the La^−^ response alone could lead coaches and researchers to exactly the opposite conclusion. It would therefore seem evident that none of the single cited parameters effectively reflects physiological strain during intermittent exercise. Despite this complex physiological picture, ventilatory parameters showed similar responses in the four investigated exercise protocols. While it is beyond the scope of this study to speculate on the mechanisms underlying such response, it is interesting to note that the common denominator between the protocols investigated seems to be a similar overall effort. In this view, it is even more interesting to consider that, beyond showing similar between-protocol responses, respiratory frequency showed an almost linear increase over time, as well as a nearly perfect relationship with RPE in all the experimental protocols. These data suggest that respiratory frequency reflects physiological strain irrespective of the CON or INT nature of the exercise.

Together with V_E_, f_R_ is the physiological parameter that accounts for the greatest source of variance in RPE during continuous exercise [28]. In addition, f_R_ and RPE respond in a similar way to some experimental interventions such as prior exercise-induced muscle damage or fatigue [29], [30] and exposure to a hot environment [31]. However, to the best of our knowledge, this is the first study that shows a strong relationship between the two parameters during intermittent exercise. While RPE is a widely recognized marker of intensity and homeostatic disturbance during exercise [32], the practice of using f_R_ as an exercise monitoring tool is not common, although promising. As a matter of fact, the linear relationship between f_R_ and RPE obtained in this study allowed us to put f_R_ values into an effort perspective (Figure 4). For instance, values of 80% and 88% of the f_R_ maximum value would correspond to an exercise effort perceived as hard and very hard, respectively. While it could be worthwhile obtaining an individualized relationship for each athlete, the association provided here can be used as a general guide for monitoring exercise sessions of both continuous and intermittent nature. Despite the similarities between RPE and f_R_ responses, they are two distinct parameters that could be used together for a psychophysiological understanding of exercise-induced fatigue. Nevertheless, one of the advantages in monitoring f_R_ over RPE is that it is an objective physiological parameter that can be measured continuously during exercise. RPE, on the other hand, is collected at discrete points in time and requires subjects to report their perception of effort, which is less practical, especially during competitions. Given the accuracy of relatively unobtrusive portable devices registering f_R_ [33], data provided in this study pave the way to possible utilization of f_R_ as a monitoring tool for physiological strain in the field, both during training and competition. This could be of particular value for exercises of an intermittent nature.

## Conclusions

The present study indicates that overall effort and total duration of exercise are two critical parameters that should both be controlled when comparing continuous with intermittent exercise protocols. On an “isoeffort” and “isotime” basis, the work-to-rest ratio manipulation affects physiological responses in a different way from what previous studies with traditional methods of comparison report [11], [24]–[26]. Despite a complex physiological framework, ventilatory parameters showed similar between-protocol responses. In particular, respiratory frequency showed an almost linear increase over time as well as a nearly perfect relationship with RPE in all the experimental protocols. These data suggest that, during intermittent exercise, respiratory frequency reflects physiological strain better than VO_2_, HR and blood lactate.
